# A high-throughput imaging and nuclear segmentation analysis protocol for cleared 3D culture models

**DOI:** 10.1038/s41598-018-29169-0

**Published:** 2018-07-24

**Authors:** Molly E. Boutin, Ty C. Voss, Steven A. Titus, Kennie Cruz-Gutierrez, Sam Michael, Marc Ferrer

**Affiliations:** Division of Preclinical Innovation, National Center for Advancing Translational Sciences (NCATS), National Institutes of Health, 9800 Medical Center Drive, Building B, Rockville, Maryland 20850 USA

## Abstract

Imaging and subsequent segmentation analysis in three-dimensional (3D) culture models are complicated by the light scattering that occurs when collecting fluorescent signal through multiple cell and extracellular matrix layers. For 3D cell culture models to be usable for drug discovery, effective and efficient imaging and analysis protocols need to be developed that enable high-throughput data acquisition and quantitative analysis of fluorescent signal. Here we report the first high-throughput protocol for optical clearing of spheroids, fluorescent high-content confocal imaging, 3D nuclear segmentation, and post-segmentation analysis. We demonstrate nuclear segmentation in multiple cell types, with accurate identification of fluorescently-labeled subpopulations, and develop a metric to assess the ability of clearing to improve nuclear segmentation deep within the tissue. Ultimately this analysis pipeline allows for previously unattainable segmentation throughput of 3D culture models due to increased sample clarity and optimized batch-processing analysis.

## Introduction

The high attrition rate of drugs in clinical trials^1^ is motivating a paradigm shift in preclinical drug discovery and development from traditional two-dimensional (2D) monolayer cell cultures to three-dimensional (3D) cell cultures and tissue models^2,3^. Unlike in 2D, cells cultured in 3D make cell-cell contacts in all dimensions, adopt complex organizational structures with other cell types and extracellular matrices, and experience relevant oxygen and nutrient gradients, all of which lead to more *in vivo*-like gene expression and cell behavior^4,5^. 3D models have been found to be more predictive of *in vivo* toxicity^6–8^, and efficacy^9^, demonstrating the promise of these models in drug development. By providing an *in vivo*-relevant test platform, 3D models have enormous potential to revolutionize the field of drug discovery.

For 3D culture systems to support drug discovery, the processes of culture production, image acquisition, and cellular phenotype analysis must be made high-throughput compatible. The most commonly used high-throughput 3D culture system is the spheroid model, in which cells are forced to aggregate into clusters using round-bottom ultra-low attachment (ULA) wells, agarose-coated microwells, or hanging drops^10^. High-throughput image analysis methods for spheroids generally rely on whole-spheroid fluorescent analysis of traditional live/dead stains^11–13^, or brightfield analysis of spheroid size as a measure of compound cytotoxicity^2,13–16^ and matrix invasion as an indicator of drug antimetastatic effects^15^. While these analysis methods provide useful information, they do not provide detailed biological or positional information of cells within spheroids. Fluorescence confocal microscopy can be used to obtain higher resolution phenotypic information. Unfortunately, light scattering prevents confocal imaging beyond a depth of around 50 µm in tissues, which limits the study of inner tissue cellular phenotypes. Optical clearing protocols have been developed to reduce light scattering and enable fluorescent imaging deep within tissues^17–22^, although application of these protocols in high-throughput environments has been limited^23,24^. Assuming the translation of clearing protocols to high-throughput environments, the challenge remains to segment and quantitate the acquired 3D image data at a cellular resolution level and in a high-throughput manner.

The ability to resolve and segment individual cell nuclei in 3D culture models is a necessary prerequisite for performing high spatial resolution measurements and quantitation of microenvironment effects. Quantitation of microenvironment effects in 3D cultures is essential, as cells within tissues may have different gene expression profiles, proliferation rates, viability, and drug responses^10,25^. Available software packages cannot perform true volumetric 3D high-content segmentation analysis in a high-throughput manner^26^. Custom methods therefore need to be developed to provide accurate segmentation of nuclei within 3D culture models^27–29^, however, existing methods rarely have the throughput necessary for application in the field of drug discovery. Herein we present a protocol for high-throughput nuclear segmentation in spheroid models. We utilized optical clearing to improve deep tissue resolution and enable acquisition on a high-content confocal microscope. The segmentation protocol can be run in batch for whole well plates and in parallel for individual spheroids. We demonstrate utility of this protocol by segmenting spheroid nuclei, identifying fluorescent subpopulation percentages, and assessing clearing and segmentation success. This workflow has the ability to change the field of 3D high-throughput analysis by enabling the acquisition of nuclear resolution information in 3D samples.

## Results

### Optical clearing enabled visualization of inner spheroid cell layers

**Figure 1:**
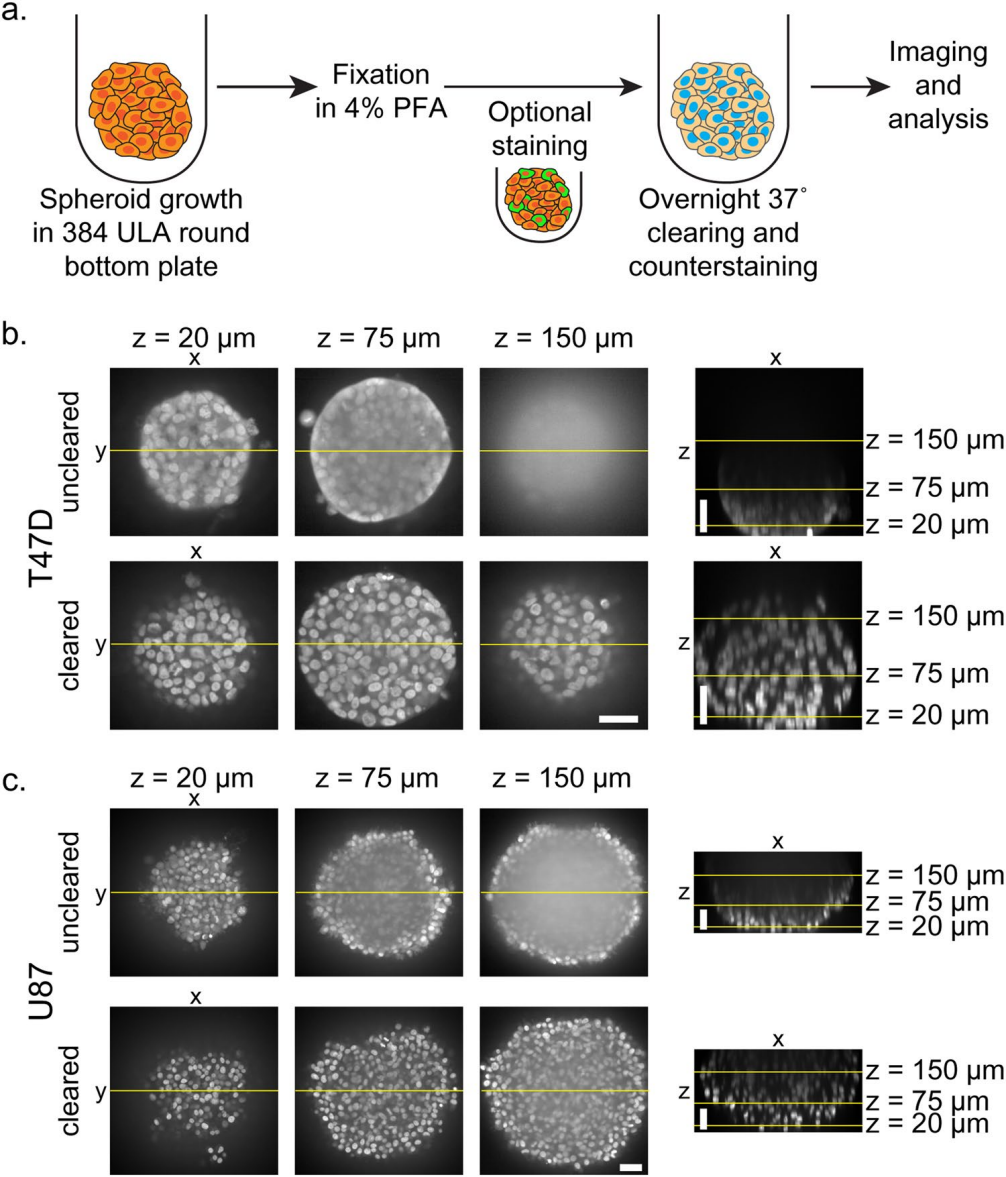
Application of optical clearing in T47D and U87 cell spheroids. (**a**) Protocol for spheroid growth, fixation, and clearing. Images of 3 DIV 500 cell T47D (**b**) and U87 (**c**) spheroids: optical slices at z-depths of 20, 75, and 150 µm into the spheroid and an orthogonal view for both uncleared and cleared samples. Yellow lines identify the corresponding cut region between the xy and orthogonal xz views. All scale bars are 50 µm.

The breast carcinoma T47D and primary glioblastoma U87 cancer cell lines were chosen to develop and validate the clearing/data acquisition/nuclear segmentation analysis method in spheroids. T47D and U87 spheroids were grown in 384-well round-bottom ULA plates and, for initial experiments, fixed at 3 days *in vitro* (DIV). All spheroid processing steps were performed in the original cell growth plates using automated liquid handling. A modified version of the ScaleS optical clearing protocol was performed to reduce light scattering and homogenize refractive indices within the spheroids^17^. This simplified and shortened clearing protocol consisted of an overnight incubation in ScaleS4 prior to imaging (Fig. 1a). ScaleS4 is a clearing reagent that contains sorbitol and glycerol for refractive index matching, DMSO and Triton X-100 for enhanced permeability, and urea for partial protein denaturation to decrease light scattering. We visualized cell nuclei using the Hoechst 33342 counterstain. Hoechst was compatible with ScaleS4 and was incorporated into the overnight incubation. To balance confocal imaging quality with a high-throughput screening environment, we utilized a high-content laser-based spinning disk confocal microscope (Opera Phenix, PerkinElmer), a 20X water objective with a 640 nm xy pixel size, and a 5 µm z-step size. Of note, average T47D and U87 nuclei diameters were 11.6 ± 2.2 µm and 13.3 ± 2.2 µm, respectively, allowing a 5 µm z-step to capture each nuclei. As expected, imaging of fixed, Hoechst-counterstained spheroids in PBS demonstrated that light scattering in the 3D samples prevented resolution of individual nuclei past several cell layers (Fig. 1b,c). The overnight application of ScaleS4 increased sample transparency, enabling resolution of nuclei deep within spheroids (Fig. 1b,c). Imaging of the Hoechst channel with a 5 µm z-step size and a total depth of 300 µm took 1 hour per full 384-well plate. Importantly, clearing application did not alter spheroid size (Supplementary Fig. 1). Hoechst fluorescent intensity did not decrease as a function of distance from the spheroid edge, showing complete Hoechst penetration into the spheroid (Supplementary Fig. 2). Additionally, Hoechst fluorescence remained relatively constant throughout z-slice depth (Supplementary Fig. 2). Qualitatively, T47D spheroids became more optically transparent and allowed for deeper imaging following clearing, compared to U87 spheroids (Fig. 1b,c).

### Nuclear segmentation protocol identified cell nuclei in cleared spheroids

**Figure 2:**
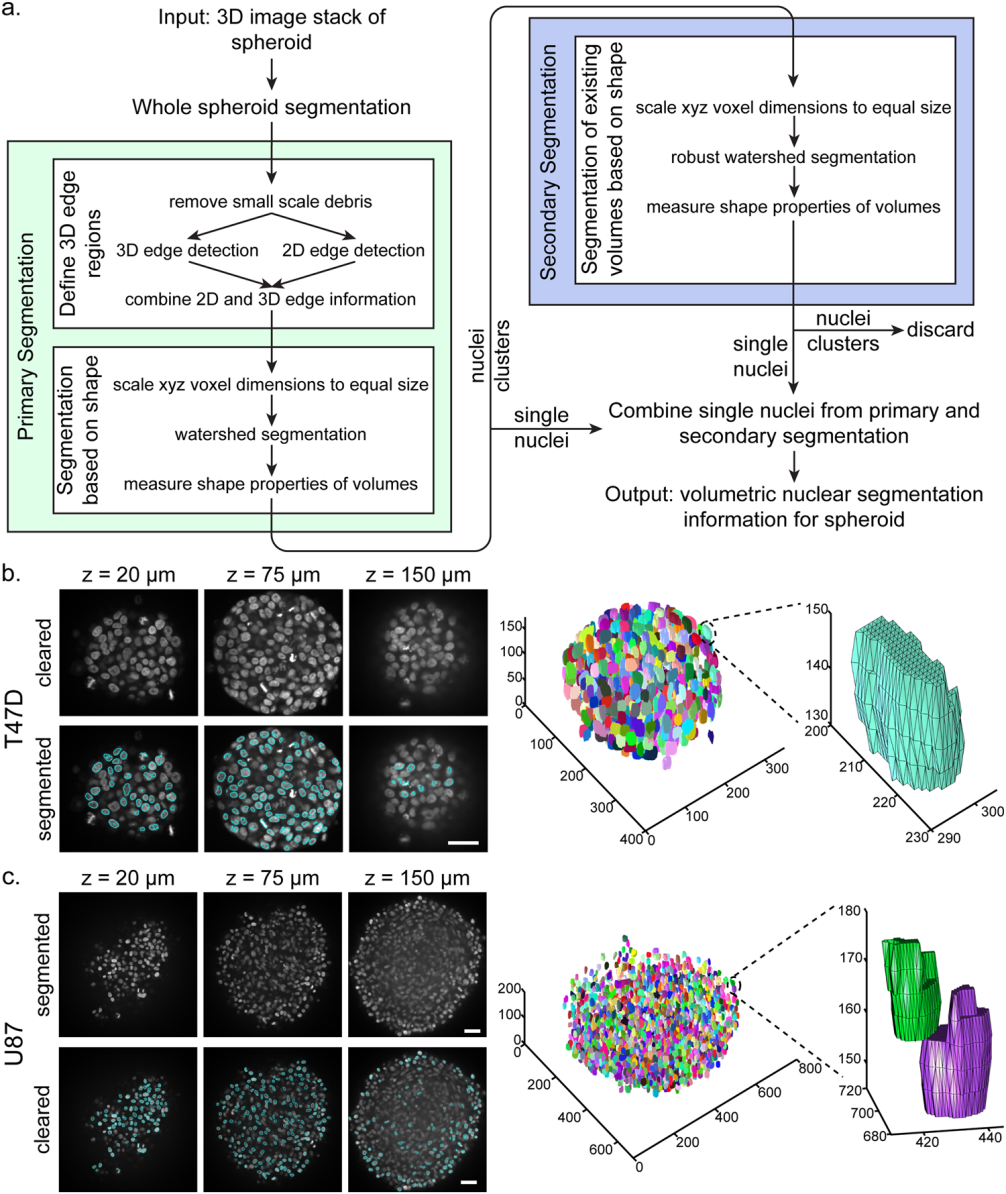
Nuclear segmentation analysis method in spheroids. (**a**) Flow chart of the segmentation analysis for an individual spheroid. Examples of T47D (**b**) and U87 (**c**) nuclear segmentation in 3 DIV 500 cell spheroids at 20, 75 and 150 µm z-depths, 3D representations of segmented nuclei, and zoomed views of specific nuclei. All scale bars are 50 µm.

The improved image quality of cleared spheroids motivated the development of new 3D image analysis techniques that were compatible with the high-throughput clearing and imaging protocols. Our primary analysis goal was nuclear segmentation in spheroids. Our analysis used raw TIFF files and was written in MATLAB (Fig. 2a). This code was developed so the input was an image folder from a 384-well plate, and the individual spheroids were analyzed in parallel on separate cores. Segmentation of the whole spheroid edge was used to crop image stacks to the spheroid size. Our primary nuclear segmentation step was biased towards under-segmentation of nuclei clusters. We performed 3D and 2D edge detection in parallel, using the higher resolution of nuclear edges in 2D images to help separate under-segmented volumes identified in the 3D edge detection. Next, a distance transform was performed such that voxel values within segmented volumes corresponded to their 3D distance from the volume edge. As clusters of nuclei have a number of maximum 3D distance regions corresponding to the number of joined nuclei, and minimum 3D distance valleys running between the joined nuclei, a watershed algorithm could be applied to separate clustered nuclei. Nuclei that remained clustered following these steps were run through a secondary segmentation that utilized a similar but more robust watershed segmentation method. The nuclear segmentation script was not modified between T47D and U87 cell types. Example images and 3D representations demonstrate segmentation of nuclei in T47D and U87 spheroids (Fig. 2b,c). Because the segmentation algorithm operated independent of nuclei intensity, we were able to segment nuclei with a range of intensities (Supplementary Fig. 2). Analysis time of a single-channel, full 384-well plate, approximately 40 GB of images, took between 5 to 6 hours on a 12-core workstation, depending on sample complexity.

In order to assess the performance of our nuclear segmentation algorithm, we generated ground truth data sets by manually segmenting nuclei of three different 3 DIV 250 cell T47D spheroids (ground truth segmentation data will be available online). These samples were chosen because their small size and young age enabled clearing and manual segmentation through the full spheroid. We then compared these ground truth sets to the automated segmentation sets, using centroid proximity to match nuclear objects^28^. We quantified the total nuclei detected in both the ground truth and automated sets, and the number of nuclei correctly detected (true positive, TP), incorrectly detected (false positive, FP), and undetected (false negative, FN). These parameters then allowed us to compute precision, which represents the number of TP out of all nuclei in the automated set, recall, which represents the number of TP out of all nuclei in the ground truth set, and F score, which is the harmonic mean of precision and recall. The average number of nuclei detected in the ground truth set and automated set was 528 and 400, respectively (Supplementary Table 1). Average TP, FN, and FP counts were 352, 176, and 48, respectively, from which the average precision, recall, and F score was calculated to be 0.88, 0.67, and 0.76, respectively. These numbers demonstrate that while our segmentation algorithm does not identify all of the nuclei in the spheroid (recall = 0.67), of the nuclei identified, the large majority are TP (precision = 0.88). Of note, for one spheroid we compared manually segmented sets that were segmented by different authors, and observed that the precision, recall, and F score was 0.82, 0.78, and 0.80, respectively. This is notable because it demonstrates that the F score, or accuracy, of our automated segmentation protocol is approaching the limit of accuracy that exists between two expert users.

### Segmentation ability depends on spheroid age, spheroid size, and cell type

**Figure 3:**
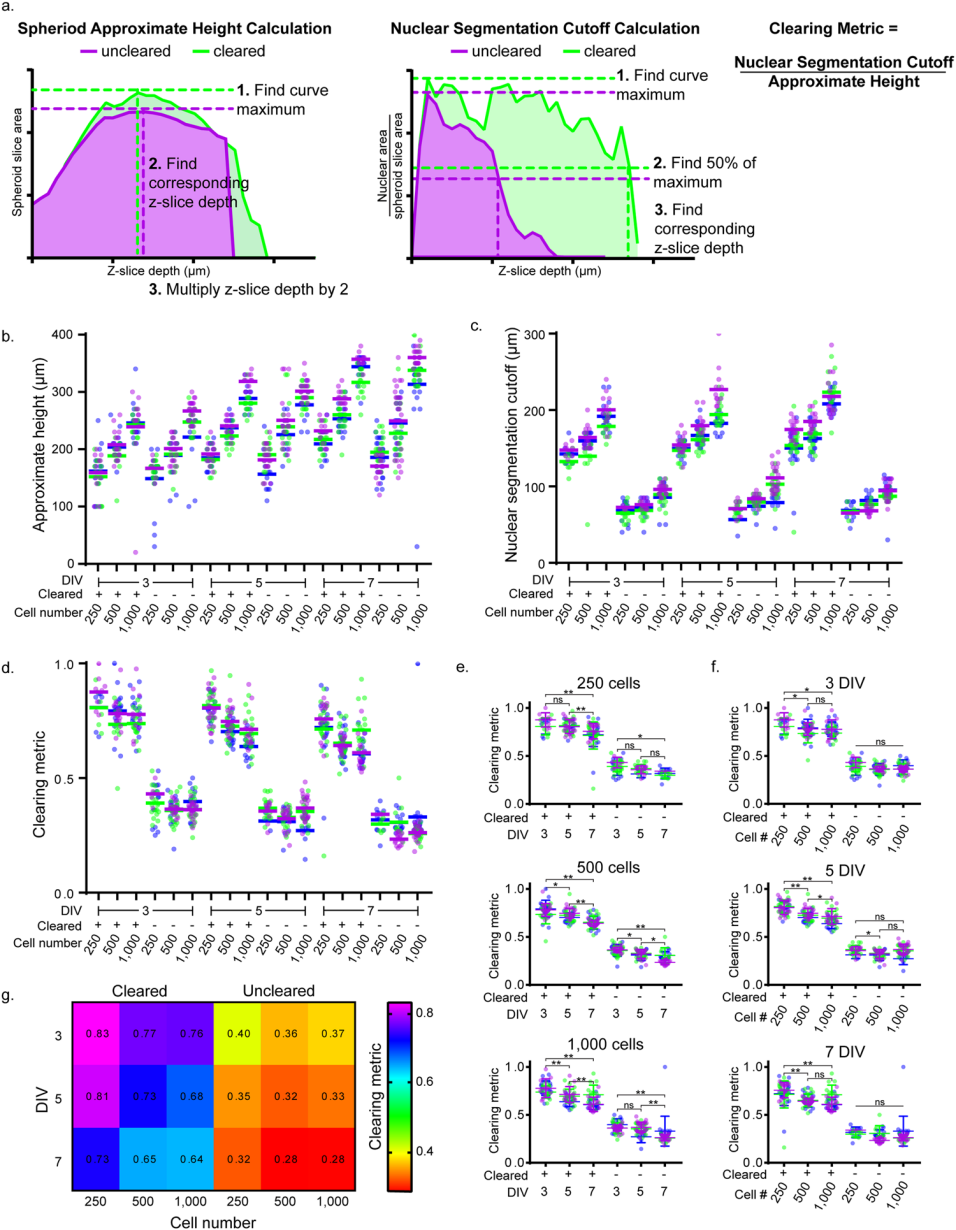
Assessment of clearing and segmentation analysis in T47D spheroids. (**a**) Approximate spheroid height, nuclear segmentation cutoff, and clearing metric calculations. Calculation steps are demonstrated using example uncleared and cleared 3 DIV 250 cell spheroids. Spheroid slice area was calculated using the segmented whole spheroid. Nuclear area was calculated by adding together the areas of each segmented nuclei within a slice. Approximate height (**b**), nuclear segmentation cutoff (**c**), and clearing metric (**d**) were calculated for three independent experiments for T47D spheroids at 3, 5, and 7 DIV and with 250, 500, 1,000 cells seeded per spheroid, for both cleared and uncleared conditions. Data points represent individual spheroids, colors represent independent experiments, and horizontal bars represent averages for independent experiments. Clearing metric data is separated to see the effect of culture time on spheroids of a single size (**e**) and the effect of spheroid size on spheroids of a single age (**f**) For (**e** and **f)** two-way ANOVA with Tukey post-hoc multiple comparisons were performed. Error bars represent SD, **P < 0.0001, *P < 0.05, ns = not significant. The number of spheroids analyzed for (**b**–**g**) can be found in Supplementary Table 2. (**g**) Heat map displays the clearing metric results, showing the average for each condition.

Qualitatively, we observed that the ability of the clearing step to improve visualization and segmentation varied between samples of different ages, sizes, and cell types. Our goal was to avoid analysis of spheroid depths that had inadequate clearing and therefore sparse nuclear segmentation, and to develop a metric to quantify the success of clearing and segmentation. While the ground truth comparison performed above is useful to assess segmentation performance, the extreme manual nature of this technique makes it infeasible for routine assessment of segmentation. In order to systematically study this behavior, we created T47D and U87 spheroids with 250, 500, or 1,000 seeded cells, and fixed these spheroids at either 3, 5, or 7 DIV. Using whole spheroid segmentation we calculated the spheroid area per z-slice, which was used to determine the approximate spheroid height (Fig. 3a). Using the nuclear segmentation we calculated the area of segmented nuclei per slice and normalized this to spheroid slice area. By looking at the ratio of nuclear area to spheroid slice area, we could determine when nuclear segmentation breakdown occurred, which we termed the nuclear segmentation cutoff (Fig. 3a). In a solid spheroid with no light scattering, the ratio of nuclear area to spheroid slice area is expected to be constant throughout spheroid z-depth, demonstrating consistent segmentation. In realistic biological samples, this ratio will decrease through z-depth due to light scattering. We used this nuclear segmentation cutoff to crop the nuclear segmentation data for each analyzed spheroid, and prevent analysis on sparsely segmented sample regions. The nuclear segmentation cutoff is influenced by spheroid size (Supplementary Fig. 3), therefore we divided the nuclear segmentation cutoff by approximate height to arrive at a clearing metric that summarizes the success of clearing and segmentation in a spheroid (Fig. 3a).

Approximate height, nuclear segmentation cutoff, and clearing metric were calculated for 3 independent experiments (T47D Fig. 3b–g, U87 Supplementary Fig. 3, Supplementary Table 2). As anticipated, approximate spheroid height increased with seeded cell number, and the nuclear segmentation cutoff occurred at lower z-slices for uncleared spheroids than for cleared spheroids (Fig. 3a–c, Supplementary Fig. 3). We used the clearing metric to understand the role of T47D spheroid age on clearing ability, demonstrating that for 250 cell spheroids there was a significantly reduced clearing ability in 7 DIV compared to both 3 and 5 DIV (p < 0.0001), and for 500 and 1,000 cell spheroids there was a significant difference between 3, 5, and 7 DIV (Fig. 3e), with older spheroids having reduced clearing ability. We additionally used the clearing metric to understand the role of T47D spheroid size on clearing ability, demonstrating that at each time point there was a significant difference between 250 cell spheroids, and both 500 and 1,000 cell spheroids (3 DIV P < 0.05, 5 DIV and 7 DIV P < 0.0001 Fig. 3f). A heat map summarized the clearing metric across all conditions (Fig. 3g). While all experiments were performed in triplicate for both T47D and U87 cell lines, U87 spheroids often grew such that their midpoints were beyond an imageable depth, preventing the calculation of approximate spheroid height and clearing metric (Supplementary Fig. 3 and Supplementary Table 2). Because the nuclear segmentation cutoff can be calculated independent of spheroid height, this metric may be useful in evaluating U87 spheroids, or other large samples (Supplementary Fig. 3).

### Analysis method can be used to detect labeled subpopulations within spheroids

**Figure 4:**
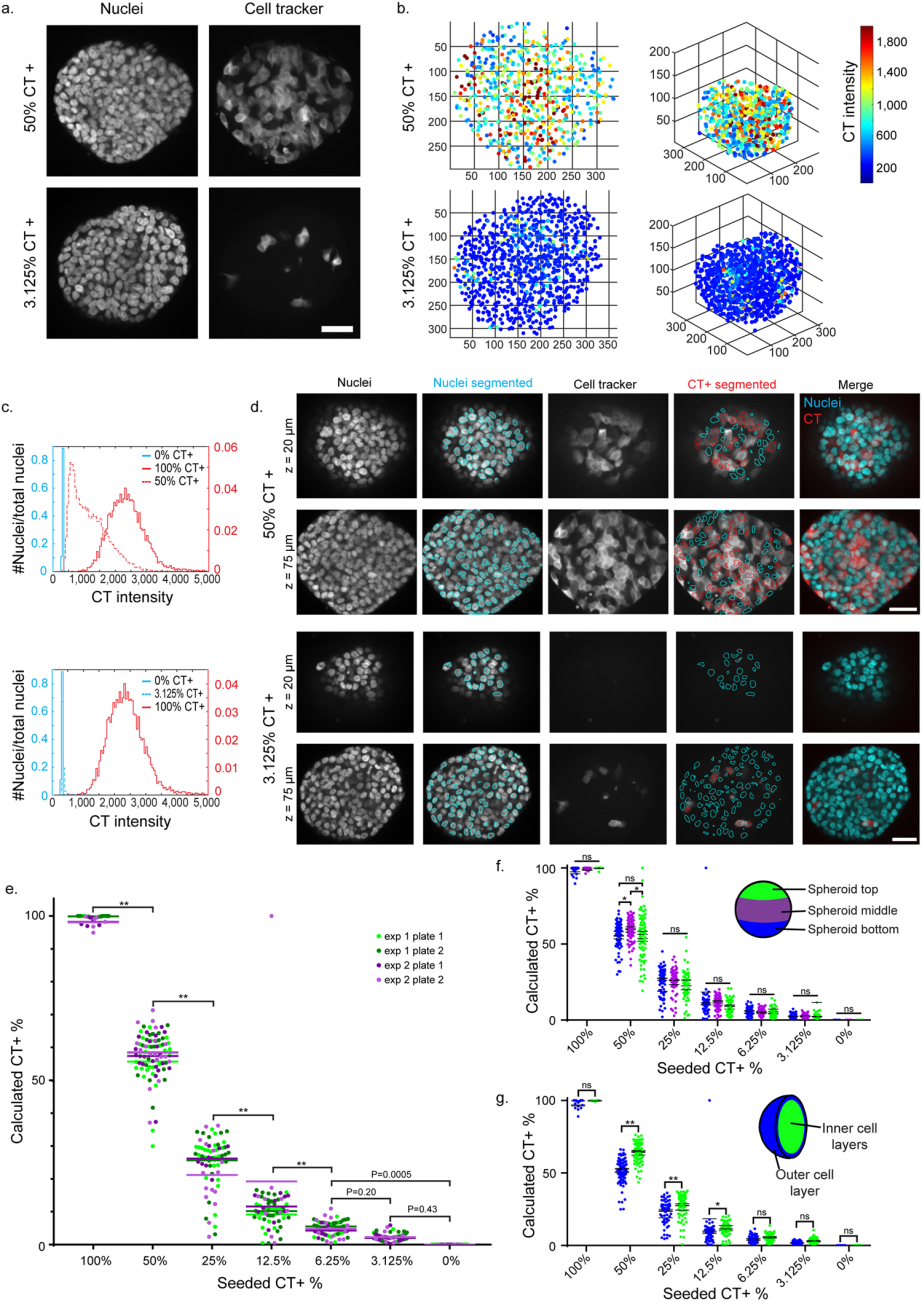
Cell tracker titration experiment demonstrated utility of segmentation analysis in T47D spheroids. (**a**) Maximum intensity projection images of a 15 µm-thick region in the center of 1 DIV 500 cell T47D spheroids with nuclear Hoechst counterstaining and 50% or 3.125% CT labeling. (**b)** Centroids of segmented nuclei within a 1 DIV 500 cell 50% or a 3.125% CT-labeled T47D spheroid. Color represents the average intensity of CT within the segmented nuclei. (**c**) Histogram depicts the CT intensity range in segmented nuclei, for control 0% CT spheroids, 100% CT+ spheroids, and 50% CT+ or 3.125% CT+. The number of spheroids analyzed was 11, 25, 22, and 23 for 100%, 50%, 3.125%, and 0% seeded CT+ conditions, respectively. (**d**) Images show segmentation examples for 50% and 3.125% CT spheroids, at 20 and 75 µm z-depths. Cyan outlines segmented nuclei and red outlines segmented nuclei determined to be CT+. (**e**) Experimental validation compared the seeded CT+% of 500 cell T47D spheroids to the calculated CT+%, as determined by the segmentation analysis. Calculated CT+% was compared between nuclei from different regions of the spheroids, spheroid bottom, middle, and top (**f**) and spheroid outer cell layer and inner cell layers (**g**) For e–g, data is displayed for two independent experiments with two replicate plates per experiment. Single points represent individual spheroids, and horizontal bars represent the average for each plate. The number of spheroids analyzed was 35, 85, 67, 66, 65, 70, and 64 for 100%, 50%, 25%, 12.5%, 6.25%, 3.125%, and 0% seeded CT+ conditions, respectively. For all statistical analysis, a two-way ANOVA was performed with Tukey post-hoc multiple comparisons **P < 0.0001, *P < 0.05, ns = not significant. All scale bars are 50 µm.

As not all cells within a spheroid were segmented in this method, we performed a controlled experiment to verify that our analysis method could accurately identify the percentage of labeled fluorescent cells in a spheroid. T47D cells were labeled with the fixable live cell dye Cell Tracker Deep Red (CT) and mixed with unlabeled T47D cells to create 500 or 1500 cell spheroids with 100%, 50%, 25%, 12.5%, 6.25%, 3.125%, or 0% CT-labeled cells. To minimize potential complications from CT-induced variations in doubling time or dye transfer between cells, spheroids were fixed 24 hours after seeding, and then taken through the clearing/Hoechst, imaging, and analysis pipeline. CT was compatible with the clearing protocol and can be seen labeling populations of T47D cells by confocal imaging (Fig. 4a). After performing the nuclear segmentation, and nuclear segmentation cutoff analysis described above, CT intensity within segmented nuclei was determined (Fig. 4b). We then created histograms depicting CT intensity within segmented nuclei (Fig. 4c and Supplementary Fig. 4) to assist in the establishment of intensity thresholds for CT labeling. As expected, the intensity histograms of positive, 100% labeled, and negative, 0% labeled, control spheroids had no intensity overlap. After mixing labeled and unlabeled cells, we observed that maximum fluorescence of labeled cells decreased and minimum fluorescence of unlabeled cells increased, relative to controls, presumably due to light scattering within the samples (Fig. 4c). The intensity threshold for determining CT+ cells was set on a per plate basis using the lower tail of the positive control spheroid histogram. Following nuclear segmentation, we applied this threshold to determine CT+ cells in a post-segmentation protocol (Fig. 4d). We performed two independent experiments, two plates per experiment, and repeated this for both 500 and 1500 cell spheroids.

For 500 cell spheroids, the average calculated CT+ percentages were 99.02%, 57.53%, 24.68%, 12.56%, 4.74%, 3.17%, and 0% for seeded CT+ percentages of 100%, 50%, 25%, 12.5%, 6.25%, 3.125%, and 0%, respectively (Fig. 4e). For 1500 cell spheroids, the average calculated CT+ percentages were 99.66%, 56.52%, 18.97%, 6.34%, 3.18%, 1.24%, and 0.01% for seeded CT+ percentages of 100%, 50%, 25%, 12.5%, 6.25%, 3.125%, and 0%, respectively (Supplementary Fig. 4). We were able to detect statistically significant differences between most fluorescent subpopulations (500 cells Fig. 4e, 1500 cells Supplementary Fig. 4), including distinguishing 6.25% CT+ cells from both 0% (500 cells P = 0.0005, 1500 cells P < 0.0001) and 12.5% CT+ (500 cells P < 0.0001, 1500 cells P = 0.003) in 500 and 1500 cell spheroids. To ensure that the analysis algorithm was not selectively segmenting or thresholding cells depending on their location within the spheroid, we compared the calculated CT+% between nuclei segmented in the bottom, middle, and top of T47D spheroids (500 cells Fig. 4f, 1500 cells Supplementary Fig. 4). There were no statistically significant differences between calculated CT+% from different depths of spheroids for 100%, 25%, 12.5%, 6.25%, 3.125%, and 0% CT+ 500 cell spheroids. We additionally compared calculated CT+% between nuclei located in the outer spheroid cell layer and nuclei located inside spheroids (500 cells Fig. 4g, 1500 cells Supplementary Fig. 4). We observed that for 50%, 25%, and 12.5% CT+ 500 cell spheroids there was a decrease in calculated CT+% in nuclei of the outer cell layer, compared to the inner cell layers (50%, 25% P < 0.0001, 12.5% P < 0.05). Because proliferative cells are typically located in the outer layer of T47D spheroids^25^, we hypothesized that this decrease in calculated CT+% of outer layer cells is due to decreased CT fluorescence intensity in labeled cells that have undergone mitosis.

We additionally used this analysis pipeline to investigate the effects of clearing on T47D-GFP endogenous GFP fluorescence intensity, and found that while clearing reduced GFP fluorescence intensity (P < 0.0001), the percent of GFP+ cells was not changed following clearing application (Supplementary Fig. 4).

### Analysis method can be used to detect changes following drug treatment

**Figure 5:**
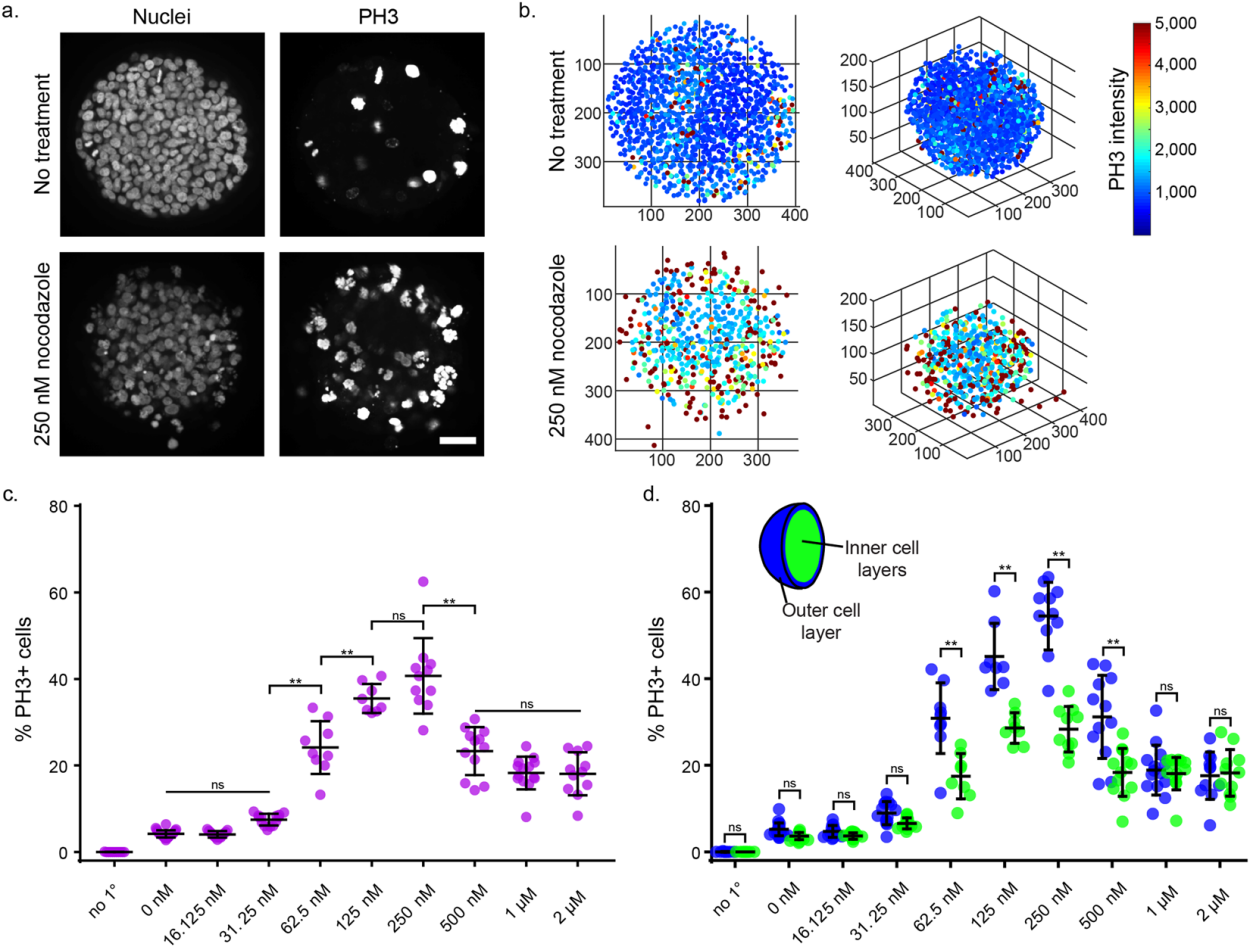
PH3 immunostaining, clearing, and segmentation analysis in nocodazole-treated T47D spheroids. (**a**) Maximum intensity projection images of a 15 µm-thick region in the center of 5 DIV 500 cell untreated and 250 nM nocodazole-treated T47D spheroids with nuclear Hoechst counterstaining and PH3 immunostaining. Image brightness is adjusted to visualize staining. (**b)** Centroids of segmented nuclei within a 5 DIV 500 cell untreated and 250 nM nocodazole-treated T47D spheroid. Color represents the average intensity of PH3 immunostaining within the segmented nuclei. (**c)** Percent of PH3+ cells following treatment with nocodazole. Control with no primary antibody was maintained (no 1°). (**d)** Percentage of PH3+ cells was compared between nuclei located in the outer cell layer and the inner cell layers. For (**c** and **d**) two-way ANOVA with Tukey post-hoc multiple comparisons was performed. Error bars represent SD, **P < 0.0001, ns = not significant. Single points represent individual spheroids. The number of spheroids analyzed was 11, 26, 13, 14, 9, 8, 11, 12, 14, and 11 for no 1°, 0 nM, 16.125 nM, 31.25 nM, 62.5 nM, 125 nM, 250 nM, 500 nM, 1 µM, and 2 µM conditions, respectively.

To demonstrate utility of the clearing and segmentation protocol in samples with more biologically-relevant fluorescent cell populations, we treated T47D spheroids with the antineoplastic drug nocodazole and performed immunostaining for the mitotic marker phospho-histone H3 (PH3). Nocodazole typically interferes with microtubule stabilization and arrests cells in mitosis^30,31^. We treated 500 cell T47D spheroids at 3 DIV with an eight-point dilution of nocodazole from 2 µM to 15.6 nM for 48 hours. Following fixation at 5 DIV, spheroids in 384-well plates were immunostained for PH3, and then brought through the clearing, segmentation, and post-segmentation protocols. PH3+ cells were found throughout the spheroids (Fig. 5a). Following nuclear segmentation, the PH3 intensity within each segmented nuclei was determined (Fig. 5b). Using the intensity threshold application in post-segmentation analysis, the percent of PH3+ cells across treatment conditions was calculated (Fig. 5c). T47D cells were found to have a biphasic dose curve, with a maximum percent of mitotic cells at 250 nM (Fig. 5c). Most cell types continue to be arrested in mitosis at increasing nocodazole concentrations. Interestingly, T47D cells grown in 2D have a similar atypical response to nocodazole, wherein at high nocodazole concentrations cells undergo arrest in G_1_ and G_2_ instead of mitosis^32^. This atypical response, shared by several cancer lines, is correlated with a p53-independent increase in p21 and thought to be important in understanding mitotic checkpoints in cancer^32^. To our knowledge, this is the first study to document the effect of nocodazole on T47D cells in 3D culture.

In untreated control spheroids there was a higher percent of PH3+ cells in the outer cell layer compared to the inner cell layers (Supplementary Fig. 5, P < 0.0001), supporting our earlier hypothesis that an increased number of proliferative cells was located in the outer cell layer (Fig. 4g). To confirm that the staining pattern observed was not due to poor antibody diffusion into the spheroid center, we immunostained spheroid cryosections (Supplementary Fig. 5), and observed the same patterning of increased PH3+ cells in the spheroid exterior. Looking across nocodazole-treated conditions we found that an increased PH3+% was observed in the outer cell layer at several treatment concentrations (Fig. 5d). This experiment highlights the ability of 3D environments to expose important cell behaviors that can only be observed by the addition of a third culture dimension.

## Discussion

In the present study we described a high-throughput pipeline for spheroid optical clearing and nuclear segmentation. Cumulatively, for the experiments presented herein, approximately 3,000 individual spheroids, 558,000 files and 960 GB of data, were segmented and analyzed. Our clearing protocol is designed to be a single step performed by automated liquid handling in 384-well plates, and was compatible with all three fluorescent proteins tested. The segmentation protocol is run in batch on full plates without user intervention. Post-segmentation analysis protocols can be customized for specific user requirements. We have shown utility of this protocol in spheroids made from both breast and brain cancer cell lines, which is important as clearing and segmentation can be affected by cell type differences such as extracellular matrix proteins and nuclear morphology. Additionally, we have demonstrated the ability of the segmentation algorithm to identify subpopulations of fluorescent cells inside spheroids, using a fixable cell tracking dye, endogenous fluorescence, and immunofluorescent staining.

Each step of our workflow was optimized for compatibility with a high-throughput environment. For optical clearing, we simplified the 72 hour, 7 exchange water-based ScaleS protocol^17^ into an overnight incubation in a single reagent, ScaleS4, which could be added by automated liquid handling. This modification shortened the protocol from the typical clearing timeframe of multiple days-weeks^17–21^, to a timeframe compatible with a high-throughput environment. For imaging, the Opera Phenix high-content confocal microscope offered the desired balance of sample resolution and imaging time. We used a 20x water objective because it afforded excellent resolution of sample nuclei. A z-step size of 5 µm achieved a balance between a small enough step size for accurate segmentation and a large enough step size for an acceptable imaging time. Imaging of a full 384-well plate took between 1–2.5 hours, depending on the number of fluorescent channels, exposure times, and z-stack size. The current method could be used as a full screen, or, to manage the large amount of image data generated, as a follow up to a first screen that identified samples of interest using sparse or single z-slice images.

The high-throughput nature of the nuclear segmentation protocol was possible due to several factors. The MATLAB analysis was written such that a whole 384-well plate could be run at one time, and such that individual spheroids could be analyzed in parallel on different cores. Several prior analyses have utilized batch or parallelized approaches to increase throughput in less complex 3D culture models, however the analysis of large numbers of samples has not been demonstrated^33,34^. In support of further increasing throughput, the initial testing of our algorithm in the MATLAB Distributed Computing Environment running on hundreds of cloud-based parallel cpu workers indicates that parallel processing is efficient when performed on a large scale (data not shown). Additionally, the segmentation algorithm was optimized for the chosen imaging parameters, most notably a sparse z-step of 5 µm. Several studies have performed nuclear segmentation in 3D culture models. Rajasekaran *et al*. imaged embryonic tissues on a traditional confocal microscope, after which 2D nuclear segmentation was performed on slices, which were subsequently joined in 3D^27^. Schmitz *et al*. imaged optically-cleared spheroids on a light-sheet microscope, and performed a classic marker-based watershed segmentation analysis^28^. One notable difference between our algorithm and these two studies is the z-slice thickness used. While these existing studies use z-slice thicknesses of 1.75 µm^27^ and 1.2 µm^28^, our algorithm was optimized to work on image stacks with a relatively sparse z-sampling interval of 5 µm, which increased both our imaging and segmentation throughput. Our segmentation protocol yielded an F score of 0.76, slightly lower than the aforementioned studies^27,28^, nevertheless our algorithm was sufficient to enable discrimination between differentially labeled subpopulations in a high-throughput fashion. The high-content confocal reader utilized in this study also allowed us to improve imaging throughput, most significantly relative to light-sheet microscopy, which requires individual sample manipulation^35^.

Two important novel aspects of the described analysis are the establishment of a nuclear segmentation cutoff and the assessment of clearing and segmentation success. This cutoff ensures that data analysis is only performed on sample regions where the segmentation algorithm is performing in an optimal manner. Deep within samples, as clearing and segmentation break down, often nuclei on the outer spheroid edges are more in focus and therefore preferentially segmented over inner nuclei, which may bias results in some experiments. The nuclear segmentation cutoff is essential because it prevents analysis in these spheroid regions which are sparsely sampled. Using the clearing metric we demonstrated that clearing and therefore segmentation ability differs depending on sample age and size. The influence of biological components on light scattering and the refractive indexes of different cellular compartments has been studied^36^, but their impact on the ability to perform nuclear segmentation has never been quantified. This metric facilitates the comparison of clearing reagents, which is necessary in the initial reagent choice, as the optimal clearing reagent will differ depending on tissue composition.

This protocol enables the study of nuclei within spheroids, and can be used to evaluate nuclear fluorescent intensity, shape, and positional information. The techniques described herein can be applied to a multitude of experiments which use fluorescent labels, including both endogenous and immunofluorescent labels. For example, this pipeline could be used to study transcription factor localization, promoter activity, histone proteins, DNA damage^37^, and nuclear morphology and aspect ratio, all of which help inform culture health, organization, and differentiation state. This pipeline also enables the visualization of fluorescently-tagged small molecules in 3D culture models, which could have broad therapeutic applications in studying small molecule localization and efficacy. Future work will expand this protocol to study non-nuclear cellular fluorescence, enabling the study of cytoplasmic fluorescence localization. Spheroids were chosen here as an example 3D culture for simplicity, but these techniques can be widely applied to different 3D culture models, including organoids and bioprinted models. As most organoid studies currently use low-throughput histological sectioning techniques to study cellular organization and differentiation, the ability to apply this pipeline to organoids opens up new avenues to study disease progression and treatment.

In conclusion, we report the first high-throughput-compatible protocol to combine clearing, high-content imaging, and analysis of spheroids. This protocol enables high-throughput drug discovery research on 3D culture models at a nuclear resolution level, opening up new opportunities in assay development and revolutionizing the field of 3D image analysis.

## Methods

### Cell culture experiments

#### Spheroid seeding and fixation

Human glioblastoma cell line U87-MG (U87, ATCC HTB-14) and human breast cancer cell line T47D-GFP (T47D, Cell Biolabs AKR-208) were cultured in standard ATCC or Cell Biolabs recommended cell culture medium. Cells were regularly mycoplasma tested and T47D cells were STR profiled. All spheroids were created by seeding a specified number of monodispersed cells in 384-well ultra-low attachment (ULA) round-bottom plates (Nexcelom ULA-384U). For studies on spheroid size and age, 250, 500, and 1,000 cells were seeded per spheroid, and plates were fixed at 3, 5, and 7 days *in vitro* (DIV). Adhesive plate sealing tape was used to prevent media evaporation over the time course of the experiments.

Sample fixation was performed by adding an equal volume of paraformaldehyde (PFA) in phosphate-buffered saline (PBS) to culture medium to create a final concentration of 4 v/v% PFA, incubating at room temperature for 30 minutes, and washing twice with PBS. Liquid additions were performed on a Biotek MicroFlo Select and liquid aspirations were performed on a Biotek ELx405 Select CW. Samples were maintained at 4 °C until subsequent processing.

#### Cell Tracker labeling

Cell Tracker (CT) Deep Red Dye (C34565, Thermo Fisher Scientific) was used according to manufacturer instructions at a concentration of 250 nM in serum-free medium. T47D cells were trypsinized, resuspended in CT-containing medium, and incubated at 37° for 45 minutes. Following incubation, cells were washed twice, incubated for 15 minutes to remove residual CT, and resuspended for counting. Spheroids with 500 and 1500 cells were seeded, with a CT-labeled population of 100%, 50%, 25%, 12.5%, 6.25%, 3.125%, and 0%. Spheroids were seeded and fixed as described above; fixation was performed at 24 hours.

#### Drug treatment and immunostaining

The drug nocodazole (M1404, Sigma-Aldrich) was used to alter the mitotic profile of T47D spheroids^30,31^. T47D cells were seeded in 384-well plates at 500 cells/well, treated with nocodazole at 3 DIV for 48 hours, and fixed at 5 DIV, as described above. Nocodazole was solubilized in dimethylsulfoxide (DMSO), and an eight-point dilution was performed from 2 µM to 15.6 nM concentration. Vehicle-only no treatment conditions were maintained as controls.

An immunostaining protocol was developed for spheroids in 384-well plates. All liquid removal steps were performed on a Biotek ELx405 Select CW, and liquid addition steps were performed by multichannel pipetting to minimize losses due to dead volume. All incubation steps were performed on a shaker. Following fixation and PBS washes, PBS was removed and blocking solution, consisting of 5 v/v% normal goat serum, 2 w/v% bovine serum albumin, and 0.5 w/v% triton X-100, was added for 30 minutes at room temperature. Primary rabbit anti-phosphohistone H3 (PH3) antibody (06–570, Millipore Sigma) was diluted in PBS with 0.5 w/v% triton X-100 (PBT) at a dilution of 1:100. Spheroids were incubated in anti-PH3 overnight (~16 hrs) at 37 °C. No-primary antibody samples were used as a control for nonspecific secondary antibody binding. Spheroids underwent 1 rinse, and 2 fifteen minute washes in PBT at 37 °C. Secondary antibody goat anti-rabbit AlexaFluor 568 (A11036, Thermo Fisher Scientific) was diluted in PBT at a dilution of 1:100. Spheroids were incubated with secondary antibody for 6 hours at 37 °C. Spheroids underwent 1 rinse, and 2 fifteen minute washes in PBT at 37 °C. A second fixation step, as recommended by Hama *et al*.^17^, was performed to crosslink antibodies to spheroid tissue. Samples were fixed in 4 v/v% PFA for 30 minutes at room temperature, followed by one ten minute wash with PBT. Samples were maintained at 4 °C until subsequent clearing.

#### Spheroid cryosectioning, immunostaining, and imaging

Following fixation and wash steps, spheroids were cryoprotected by equilibrating in 15 w/v% and then 30 w/v% sucrose in PBS over 6 hours. Spheroids were embedded in Optimal Cutting Temperature compound (O.C.T.), stored at −80 °C until use, and sectioned on a Thermo Scientific CryoStar NX50 into 8 µm-thick cryosections. Immunostaining of cryosections was performed on a BOND RX^m^. Briefly, sections were blocked for 20 minutes with a 2 v/v% normal goat serum, 1 w/v% bovine serum albumin solution in PBS. Primary PH3 antibody, as above, was diluted at 1:500 in PBS and samples were incubated for 30 minutes. Following wash steps, secondary antibody, as above, was diluted at 1:500 in PBS and samples were incubated for 20 minutes. Following wash steps, samples were incubated in Hoechst at a dilution of 1:2000 for 5 minutes. Sections were mounted using Fluoromount-G and imaged on a Leica TCS SP8 Confocal Laser Scanning Microscope built on a Leica DMi8 inverted microscope with LAS X software using a 25x water objective (NA 0.95, 506375, Leica). Image stacks were taken with an optimal z-slice thickness of 0.56 µm.

#### Optical clearing

Optical clearing was adapted from the protocol developed by Hama *et al*.^17^. The clearing solution used was ScaleS4, which is composed of 40 w/v% D-(-)-sorbitol, 10 w/v% glycerol, 4 M Urea, 0.2 w/v% Triton X-100, and 15 v/v% DMSO in ultrapure water. The solution was agitated at 37 °C to mix, and stored at 4 °C until use.

Following fixation and, if applicable, immunostaining, PBS was removed and ScaleS4 solution containing the nuclear counterstain Hoechst 33342 was added (30 uL/well) using a Biotek Microflo Select. Uncleared control samples were incubated in PBS with 0.2 w/v% triton X-100 and Hoechst. Plates were incubated in clearing solution overnight (~16 hrs) at 37 °C on a shaker.

### Imaging

Following clearing, 384-well plates with spheroids were imaged on an Opera Phenix high-content microscope (PerkinElmer) using a 20x water objective (NA 1.0, HH14000421, PerkinElmer), with 2x digital camera pixel binning, which yields a xy pixel size of 640 nm. Image stacks were taken with 5 µm step sizes to a total depth of 200 µm for U87 spheroids and 300 µm for T47D spheroids. Excitation/emission wavelengths used were 405/435-480 for Hoechst, 488/500-550 for GFP, 561/570-630 for AlexaFluor 568, and 640/650-760 for CT Deep Red.

For time course and seeding density experiments, experiments were repeated in triplicate, with 28 T47D wells imaged per condition per experiment. Fifty-six U87 wells were imaged per condition per experiment, except experiment 1 1000 cell condition, and experiments 2 and 3 250 cell condition, for which 28 wells were imaged. For CT experiments, experiments were repeated in duplicate, with 2 plates per independent experiment. Fourteen wells were imaged for the 100% CT condition per plate, and 28 wells were imaged per plate for 50%, 25%, 12.5%, 6.25%, 3.125%, and 0% conditions. The PH3 data shown is representative of several repeated experiments. Sixteen wells were imaged per treatment condition, 14 wells for the no primary control, and 28 wells for the untreated condition.

### Nuclei Size Measurements

The average size of T47D and U87 cell nuclei in spheroids was determined by measuring the nuclei diameter in ImageJ/FIJI. For each cell type the diameter of 50 random nuclei was measured.

### Segmentation Analysis

#### Analysis protocol overview

Following imaging on the Opera Phenix, images were exported as individual 16 bitt TIFF files. We developed a customized MATLAB script that automatically analyzes both the 3D volume of each individual nucleus in the spheroid as well as the 3D structure of the entire spheroid. Versions of the custom script run in the MATLAB R2017B software environment and can take advantage of multiple cpu cores within a single Windows OS workstation PC or Unix-based High-Performance Computing Clusters. The following commercially available MATLAB Toolboxes are required: Image Analysis, Parallel Computing. The sequential broad functions of the analysis routine are as follows: (1) Organizing and loading image data from storage drive, (2) Cropping 3D volume to rough whole spheroid dimensions, (3) Defining 3D volumes for each individual nucleus, (4) Defining 3D volume for whole spheroid, (5) Calculating morphometric properties for each nuclear volume and the whole spheroid volume, and (6) Saving output data. The complete annotated MATLAB code for this algorithm is available via online repository (https://github.com/boutinme/3D-Nuc-Seg).

#### Image organization and import

Image sets from individual wells of the multiwell plate serve as the input for the main subroutine. For the experiments described here, each set contains: multiple fluorescence intensity channel images and multiple z-depth image planes. An automatic image organization and file loading steps allow straightforward and efficient parallel processing. Using functions from the commercially available MATLAB Parallel Computing Toolbox, an entire image set for each well in the plate is simply parsed to an individual CPU core within a multi-CPU core workstation and/or across worker nodes of high-performance computer cluster.

#### Spheroid identification and image cropping

After an image set is loaded, a threshold identifies the voxels in the nuclear channel image stack that are above background intensity level. A black and white morphological closing operation then connects these bright voxels into a volume that approximates the whole spheroid shape. The size of the detected volume is used to automatically determine if a spheroid is present in the well and if analysis should continue for that image set. If the whole spheroid volume is overlapping with the X-Y edge of the image stack, then the image set is automatically excluded from further steps of the analysis. The cuboid sub-region containing the spheroid (3D bounding box) is automatically cropped, and this subregion is then used for all subsequent steps of the segmentation algorithm.

#### Definition of 3D volumes for individual nuclei

After Gaussian smoothing, a combination of 2D and 3D Laplacian edge detection filters identifies the 3D boundaries of individual nuclei and clusters of nuclei. The size of the Gaussian filter is set to be large enough to minimize the influence of subnuclear features (for example dim Hoechst staining of nucleoli and bright Hoechst staining of pericentromeric chromatin), and also small enough to maintain the fluorescence intensity boundaries between individual nuclei. Our results demonstrate that a single setting for Gaussian filter can operate on different cell types and different growth conditions. As in many imaging analysis approaches, signal to noise ratio is the limiting factor for the method described here. At deep Z-planes, detected edges become weaker due to a reduction in signal to noise ratio. However, our 3D nuclei segmentation method is designed to have minimal reliance on absolute fluorescent intensities from the spheroid samples. We refer to the volumes enclosed by these edge detection filter boundaries as potential nuclear volumes (PNVs). The algorithm primarily utilizes the 3D shape of the identified PNVs to determine if these volumes correspond to single nuclei. If the PNVs are not single nuclei, the algorithm also relies on 3D shape to split the multi-nuclei clusters into distinct single nuclei volumes. The provided MATLAB code contains several conditional statements which specify the current parameters for selection of single nuclei volumes. For future, advanced methods development (studies in different cell lines, growth conditions, imaging modalities), the current conditional statement selection parameters in our MATLAB code can be manually altered or replaced with machine learning-optimized parameters. Here we describe the general principles and additional steps of this 3D nuclear segmentation approach. A distance transform produces an output image stack where each voxel value represents the 3D distance of that voxel to the edge of the PNV. Single nuclei are typically ellipsoid-shaped volumes. Ideal ellipsoid volumes contain a single local maximum distance region at the 3D center. Clusters of multiple nuclei often appear as multi-lobed ellipsoid volumes, and therefore these volumes contain multiple maximum distance regions. Within multi-nuclei clusters, the 3D junctions between individual nuclei (boundaries between touching nuclei) are output as local minimum ‘valleys’ in the distance transform values. Because the watershed transform function can use local minima regions as seeds for the separation of touching regions/volumes, the intensity complement of the distance transform stack is used in subsequent steps. After image complementation, the 3D centers of the ellipsoids are now local minima values. Small deviations from the ideal ellipsoid shape will cause additional slight local minima in the complemented distance transform stack. An H-minima transform function suppresses these slight local minima and prevents over-segmentation of true nuclear volumes (TNVs). A watershed transform is then applied to the H-minima output image stack to segment individual nuclei volumes. The properties of the watershed output volumes are measured and used to sort TNVs and residual multi-nuclei clusters. Residual cluster volumes are then re-segmented based on sequential application of distance transform, a less aggressive local minima suppression function, and watershed transform. TNVs from this secondary nuclear segmentation are combined with the TNVs from the primary nuclear segmentation to produce the set of detectable individual nuclear volumes within the spheroid. This approach provides a balance of both computational speed and robust segmentation across a variety of experimental conditions.

#### Definition of 3D volume for whole spheroid and morphometric calculations

The light scattering properties of cleared and uncleared spheroids are drastically different from each other. As a result, the fluorescence intensity signal to noise ratios for the two sample types are also widely divergent. However, individual nuclei can be robustly segmented within the shallow z-planes of both cleared and uncleared samples. Two distinct steps of the whole spheroid segmentation analysis subroutine mitigate these challenging conditions. Firstly, the strategy for shallow z-planes is simple fusion of single nuclei volumes by morphological closure to produce the whole spheroid volume. Secondly, median filtering of the nucleus channel intensity image reduces blur surrounding the whole spheroid volume, and reduces detail in the whole spheroid region. The reduction of detail is sufficient to mask individual nuclei, which facilitates the later segmentation of the whole spheroid as a single volume. Edge detection and thresholding based on the whole spheroid edge regions define the whole spheroid region. We found this to be the optimal strategy for defining the spheroid volume at deeper z-planes where segmentation of individual nuclei becomes more difficult. The outputs of shallow z-plane strategy and the deep z-plane strategy are combined to produce the whole spheroid volume. This volume is then input into a 3D distance transform to calculate the distance for every voxel in the whole spheroid volume. Additional morphometric values are calculated as described for individual experiment results.

#### Saving output data

To allow flexible and portable subsequent analysis steps, nuclear segmentation and whole spheroid analysis outputs are saved as separate.mat MATLAB files for each analyzed spheroid. This.mat file contains the location information, nuclear channel intensity, and additional channel intensity for all segmented nuclear volumes, as well as the large spheroid segmentation outputs.

### Post-Segmentation Analysis

The post-segmentation script utilizes the nuclear segmentation information from the above analysis, as well as additional user inputs for specific analysis protocols. We primarily utilized the post-segmentation script to quantify the success of clearing and segmentation, and to determine the percentage and location of fluorescently-labeled cells.

For determining success of clearing and segmentation, a clearing metric was calculated using the following process. Spheroid slice area was calculated using the size of the segmented whole spheroid. The z-slice of the maximum spheroid slice area was multiplied by two to calculate an approximate spheroid height. Spheroid height was not calculated for spheroids whose per slice area did not decay to 97% of the maximum, an indication that the middle of spheroid was not imaged. To understand how segmentation progressed through the spheroid, the area of segmented nuclei per slice was divided by the spheroid slice area. After smoothing this ratio, the z-slice of a decrease of 50% from the maximum was defined as the reliable nuclear segmentation cutoff. For spheroids whose ratio did not decrease below 50% in the imaging window, a nuclear segmentation cutoff was not reported. This cutoff was applied such that only nuclei segmented before the cutoff were used in subsequent analysis. If 50% of the maximum was not reached, then the z-slice of 10% of the maximum spheroid area was applied to crop the segmentation data. The clearing quality metric was defined as the ratio of the nuclear segmentation cutoff to the approximate height. The few cases where the clearing metric was over 1 were excluded due to misapproximation of spheroid height. The reported number of spheroids for each metric can be found in Supplementary Table 2.

To determine the percentage of fluorescently labeled cells, a simple threshold was applied. For the experiments herein, because labeled cells had fluorescence within their nuclei, the average intensity of the fluorescent label channel within a nuclear ROI was used to determine whether a nuclei was positive or negative for a given fluorophore. We analyzed where in the spheroid the positively labeled cells were located by splitting the spheroid into sections. The bottom, middle, and top of the spheroid were defined as <60 µm, 60–120 µm, and >120 µm, respectively. The outer cell layer of the spheroid was defined as within 12.8 µm of the spheroid edge, and the inner cell layers were inside of 12.8 µm from the spheroid edge.

### Ground Truth Comparison

Three whole 3 DIV 250 cell T47D spheroids were chosen for manual nuclear segmentation using a custom program. One author manually segmented all three spheroids, while a second author segmented one of the same spheroids to have a comparison between users. Once nuclei were manually segmented, the centroids of these ground truth nuclei were compared to those detected by the automated segmentation algorithm. The method previously described by Schmitz *et al*.^28^ was used to compare the data sets. Briefly, if a centroid of the automated set was found within 6 µm of a centroid of the ground truth set, this nuclei was called a TP. If multiple automated set centroids were within the specified range from a ground truth centroid, the closest was termed TP. FP was calculated by subtracting the TP from the total nuclei in the automated set, while FN was calculated by subtracting TP from the total nuclei in the ground truth set. Precision, recall and F score were calculated at shown below:

1 $$precision=\frac{TP}{TP\,+\,FP}$$

2 $$recall=\frac{TP}{TP+FN}$$

3 $$F\,score=2(\frac{precision\,\times \,recall}{precision+recall})$$

### Statistical Tests

All statistical tests were performed in GraphPad Prism 7. Details on tests performed are located in figure legends. For most grouped studies a two-way ANOVA was followed by Tukey post-hoc multiple comparisons test. All t-tests were two-tailed. All error bars were standard deviation (SD). The following nomenclature was used in all figures: **P < 0.0001; *P < 0.05; ns, not significant, P > 0.05.

### Code Availability

MATLAB code is available at https://github.com/boutinme/3D-Nuc-Seg.

### Data Availability

The datasets generated and analyzed during the current study are available in the NCATS public repository, https://tripod.nih.gov/pub/clearingdata/.

## Electronic supplementary material

Supplementary Information
