# Supplementary material for: A high-throughput imaging and nuclear segmentation analysis protocol for cleared 3D culture models
Source: Sci Rep. 2018 Jul 24;8:11135. doi: 10.1038/s41598-018-29169-0 (PMC6057966; doi:10.1038/s41598-018-29169-0)

A high-throughput imaging and nuclear segmentation analysis protocol for cleared 3D culture models

Molly E. Boutin^1*^, Ty C. Voss^1^, Steven A. Titus^1^, Kennie Cruz-Gutierrez^1^, Sam Michael^1^, Marc Ferrer^1^

^1^Division of Preclinical Innovation, National Center for Advancing Translational Sciences (NCATS), National Institutes of Health, 9800 Medical Center Drive, Building B, Rockville, Maryland 20850, USA

*Corresponding author

**Supplementary Figures**


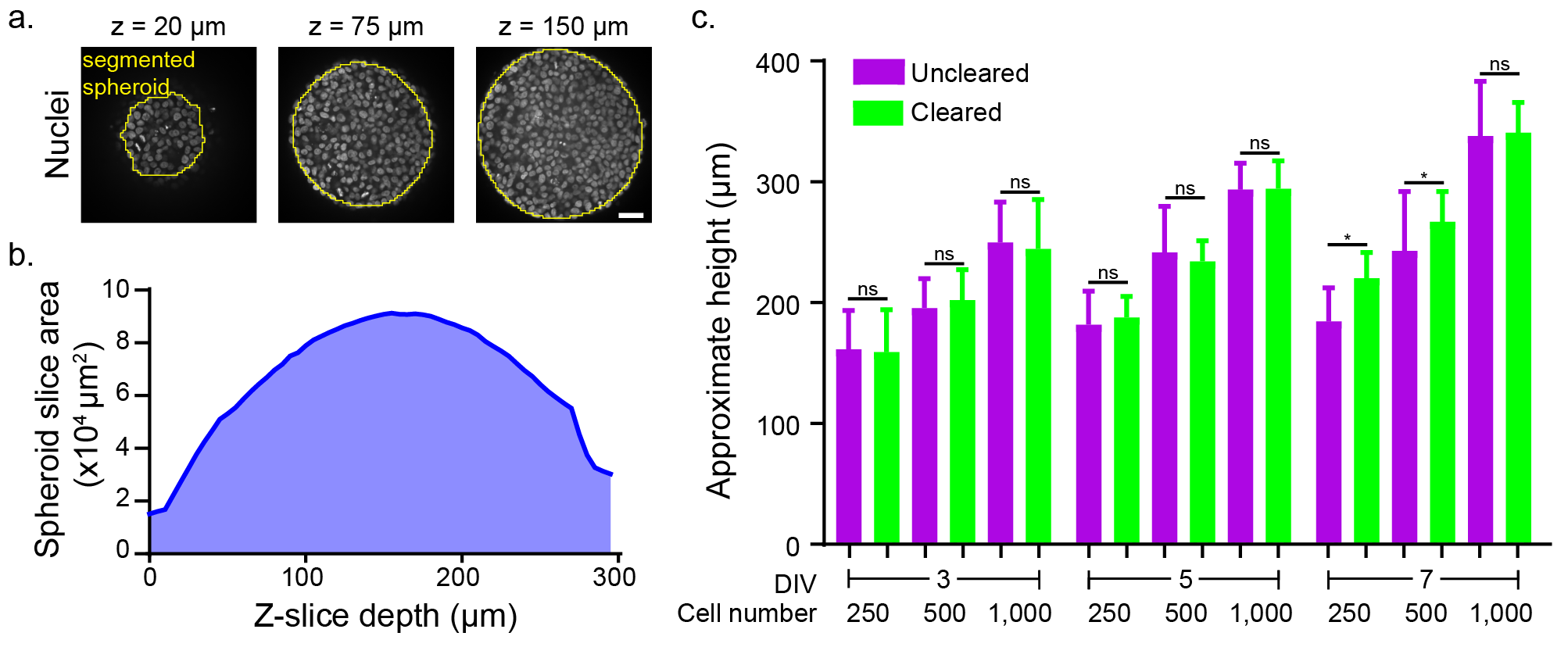
Supplementary Figure 1. Spheroid size was not altered by clearing reagent. **a.** A whole spheroid segmentation algorithm was developed to identify the spheroid edge. Example images of nuclei and the identified spheroid edge (yellow line) are shown for a 7 DIV 1,000 cell spheroid at z-depths of 20, 75, and 150 µm into the spheroid. Scale bar is 50 µm. **b.** Using the whole spheroid segmentation algorithm, spheroid slice area was calculated for all z-slice depths. Graph depicts the example spheroid shown in **a.** Approximate spheroid height was calculated by finding the z-slice depth of the maximum on the spheroid slice area curve, and multiplying by two. **c.** Approximate height was calculated for both uncleared and cleared T47D spheroids at 3 DIV and 3 spheroid sizes. Data shown is a compilation of three independent experiments. The number of spheroids analyzed, from left to right on the graph, was 49, 43, 72, 51, 64, 49, 61, 63, 61, 58, 63, 51, 74, 55, 69, 60, 50, and 70. A one-way ANOVA followed by post-hoc Tukey’s multiple comparison test was performed. Error bars represent SD, *P < 0.05, ns = not significant.


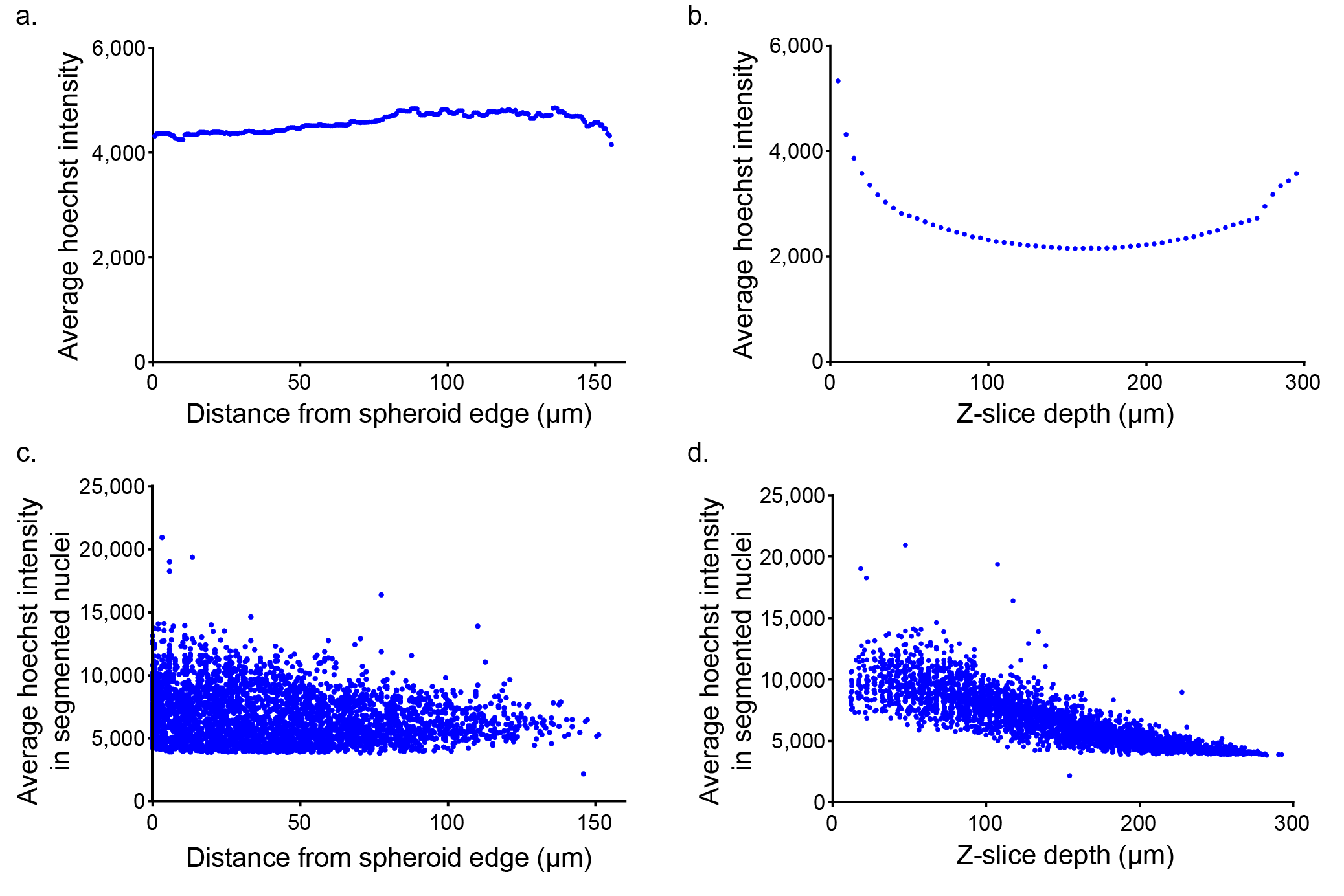

Supplementary Figure 2. Hoechst nuclear counterstain intensity within spheroids. **a.** Average Hoechst channel pixel intensity versus distance from spheroid edge, where spheroid edge is found by the whole spheroid segmentation. **b.** Average Hoechst channel pixel intensity across the z-slice depths of the whole segmented spheroid. **c.** Average Hoechst intensity in segmented nuclei versus distance from spheroid edge, where spheroid edge is found by the whole spheroid segmentation. **d.** Average Hoechst intensity in segmented nuclei across the z-slice depths of the whole segmented spheroid. For both **a-d** data is a representative graph of a 7 DIV 1,000 cell T47D spheroid. For **c.** and **d.** each data point represents a single segmented nuclei.


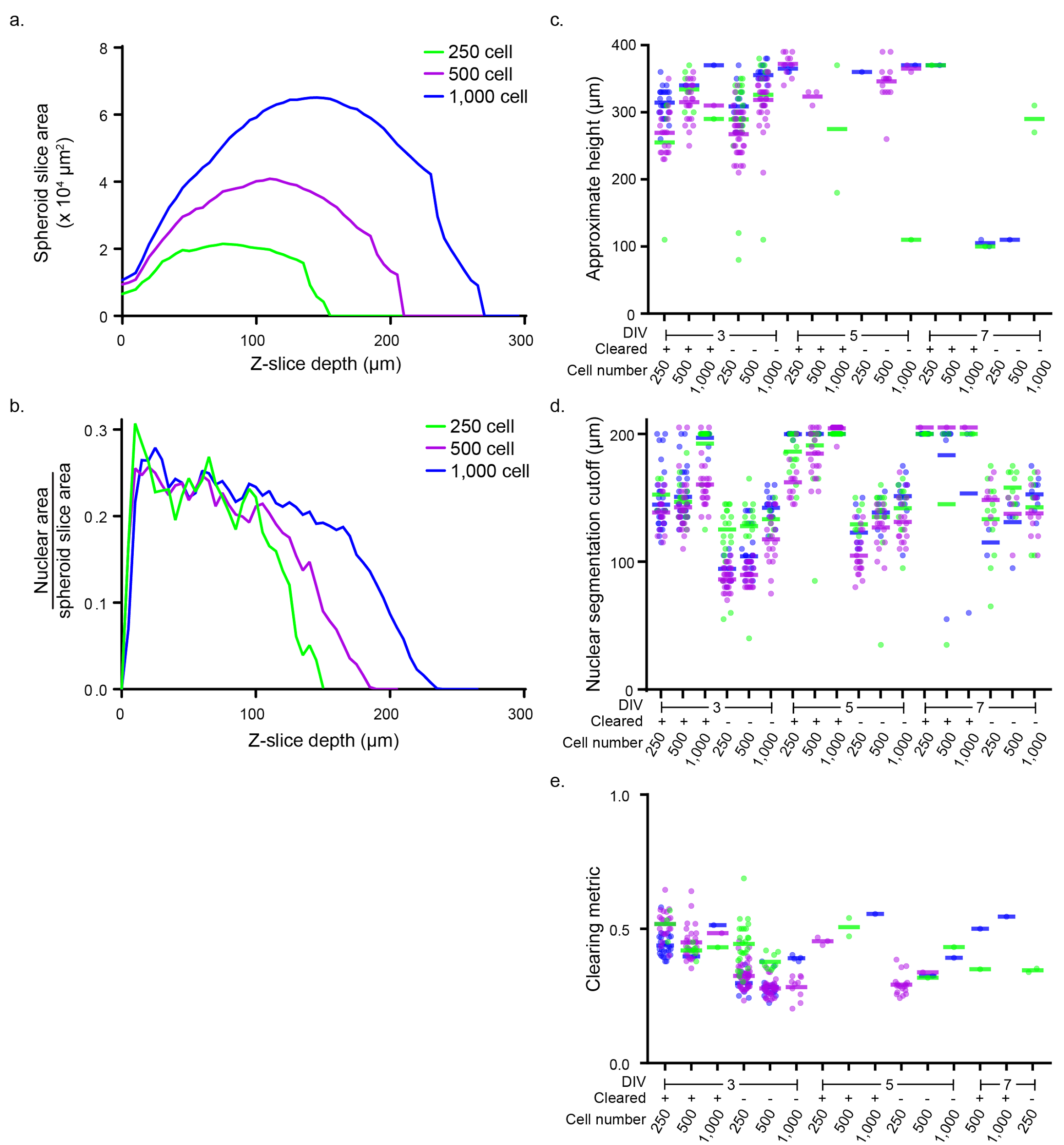
Supplementary Figure 3. Assessment of clearing and segmentation analysis in spheroids. Spheroid slice area (**a.**) and nuclear area over spheroid slice area (**b**.) graphs showing data for example for representative 5 DIV T47D spheroids with 250, 500, and 1,000 cells. Approximate height (**c.**), nuclear segmentation cutoff (**d.**), and clearing metric (**e.**) were calculated for three independent experiments for U87 spheroids at 3, 5, and 7 DIV and with 250, 500, 1,000 cells seeded per spheroid, for both cleared and uncleared conditions. Data points represent individual spheroids, colors represent independent experiments, and horizontal bars represent averages for independent experiments. The number of spheroids analyzed for **c.-e**. can be found in Supplementary Table 1.


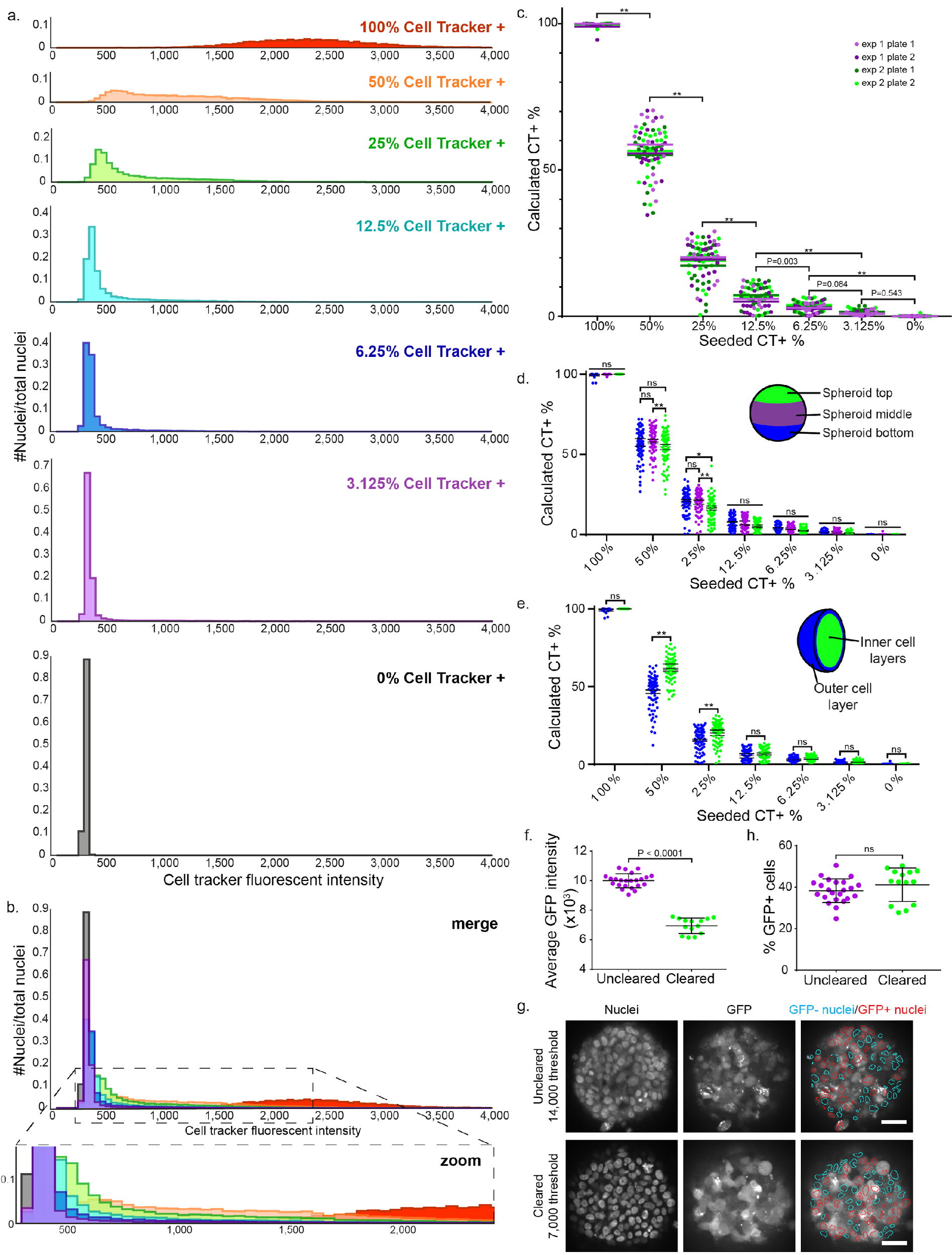


Supplementary Figure 4. Fluorescence subpopulation analysis in T47D spheroids. **a-b.** Histograms of CT 647 fluorescent intensity in 1 DIV 500 cell spheroids following nuclear segmentation analysis. Histogram analysis combines data from all spheroids of a given condition in a single experiment. Data is displayed as (**a**.) separated histograms of all conditions and (**b**.) merged views of histograms from all conditions. The number of spheroids analyzed was 11, 25, 24, 21, 20, 22, and 23 for 100%, 50%, 25%, 12.5%, 6.25%, 3.125%, and 0% seeded CT+ conditions, respectively. **c.** Experimental validation compared the seeded CT+% of 1500 cell T47D spheroids to the calculated CT+%, as determined by the segmentation analysis. Calculated CT+% was compared between cells from different regions of the spheroids, spheroid bottom, middle, and top (**d.**) and spheroid outer cell layer and inner cells (**e**.). For **c-e.** data is displayed for two independent experiments with two replicate plates per experiment. Single points represent individual spheroids, and horizontal bars represent averages for each plate. The number of spheroids analyzed was 28, 75, 72, 67, 76, 80, and 88 for 100%, 50%, 25%, 12.5%, 6.25%, 3.125%, and 0% seeded CT+ conditions, respectively. For statistical analysis, a two-way ANOVA was performed with Tukey post hoc multiple comparisons **P<0.0001, *P<0.05, ns = not significant. **f.** T47D-GFP 3 DIV 1,000 cell spheroid endogenous fluorescence intensity was compared by finding the average GFP intensity within the spheroid, as outlined by the whole spheroid segmentation algorithm. A two-tailed unpaired t-test was performed. Error bars represent SD. **g.** Example images of 3 DIV 1,000 cell cleared and uncleared spheroids, showing nuclear counterstaining and endogenous GFP. Cyan outlines segmented nuclei determined to be GFP- and red outlines nuclei determined to be GFP+, as determined by setting appropriate thresholds for uncleared and cleared samples. Scale bars are 50 µm. **h.** Percentage of GFP+ cells in 3 DIV 1,000 cell whole spheroids, as determined by post-segmentation analysis in which appropriate thresholds were set for uncleared and cleared groups. A two-tailed unpaired t-test was performed. Error bars represent SD, ns = not significant. For **f.** and **h.** data points represent individual 3 DIV 1,000 cell spheroids. Twenty-three spheroids were analyzed for uncleared, and 14 for cleared.


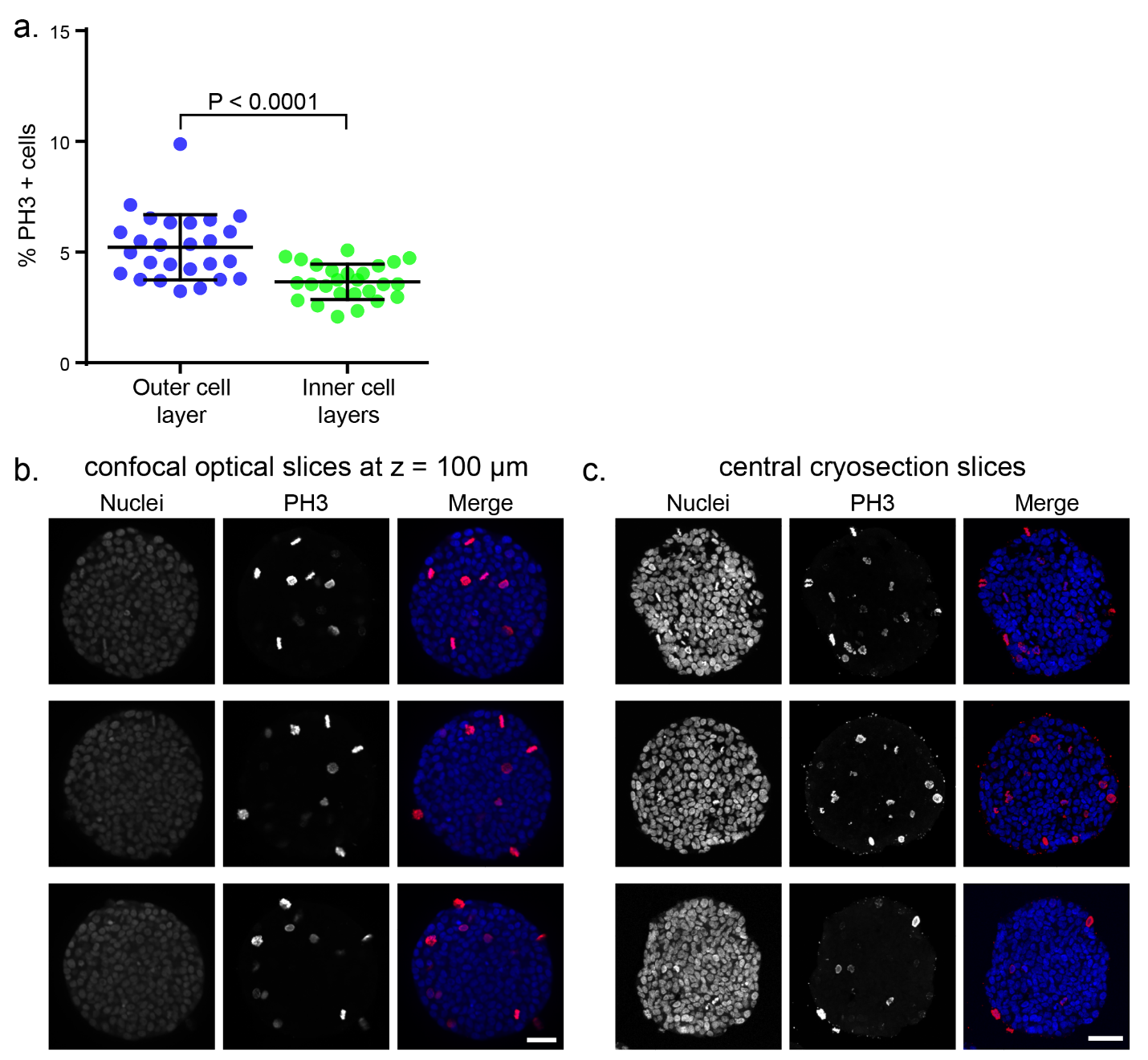


Supplementary Figure 5. Comparison of PH3+ cell location in untreated condition. **a.** Percentage of PH3+ cells was compared between nuclei located in the outer cell layer and the inner cell layers. A two-tailed unpaired t-test was performed. Error bars represent SD. Single points represent individual spheroids, 26 spheroids were analyzed. **b.-c.** To confirm the staining patterning shown in confocal optical slices (**b.**), which shows increased number of PH3 + cells towards the spheroid exterior, physical cryosections were taken and immunostained for PH3 (**c.**), which revealed a similar patterning. Scale bars are 50 µm.

Supplementary Table 1. Evaluation of automated segmentation compared to ground truth data sets. Three 3 DIV 250 cell T47D spheroids were manually segmented to acquire ground truth data sets, and the centroids of these ground truth sets were compared to the output of the automated segmentation algorithm. Details on calculations can be found in the methods section. Abbreviations: TP, true positive; FN, false negative; FP, false positive; S.D., standard deviation


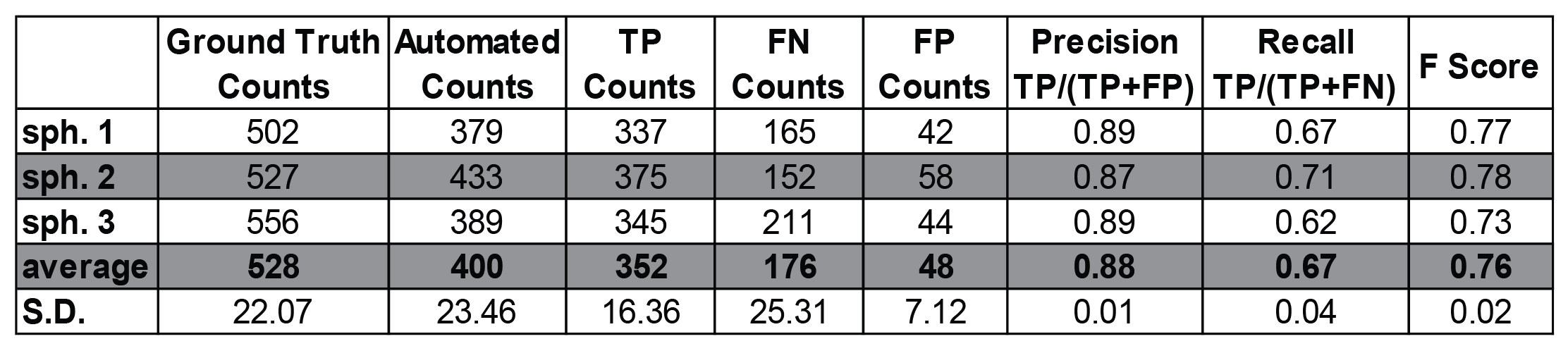


Supplementary Table 2. Numbers of T47D and U87 spheroids analyzed for clearing and segmentation assessment experiments. Shown are the number of spheroids segmented, and the number analyzed in approximate height, nuclear segmentation cutoff, and clearing metric calculations. Commas are used to separate the number of spheroids analyzed for independent experiments. The number of wells imaged per condition and the exclusion criteria for spheroids can be found in the methods section.


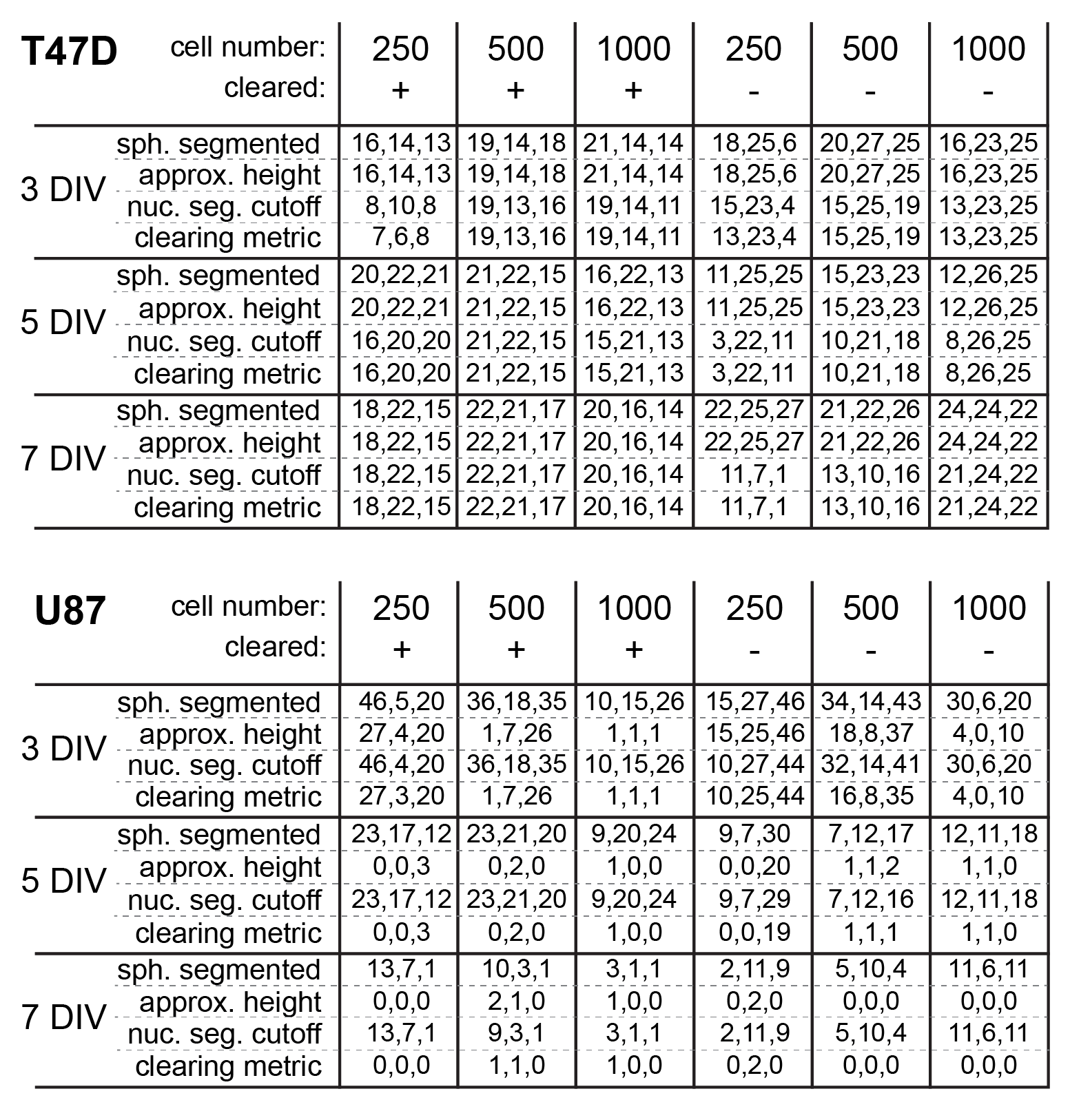

Supplement: Supplementary file 1 — Supplementary Information [file 41598_2018_29169_MOESM1_ESM.docx]
